# 1,5,7,8′,11-Penta­meth­oxy-13*H*-spiro­[dibenzo[*a*,*g*]fluorene-13,1′(4′*H*)-naphthalen]-4′-one toluene monosolvate

**DOI:** 10.1107/S160053681203824X

**Published:** 2012-09-12

**Authors:** Ryo Takeuchi, Atsushi Nagasawa, Akiko Okamoto, Noriyuki Yonezawa

**Affiliations:** a Department of Organic and Polymer Materials Chemistry, Tokyo University of Agriculture & Technology, 2-24-16 Naka-machi, Koganei, Tokyo 184-8588, Japan

## Abstract

In the title compound, C_35_H_28_O_6_·C_7_H_8_, the dihedral angle between the mean planes through the naphthalene ring systems of the dibenzo[*a*,*g*]fluorene moiety is 22.44 (3)°. The aromatic ring system of the naphthalenone unit is approximately perpendicular to the mean plane of the five-membered ring, forming a dihedral angle of 87.51 (5)°. An intra­molecular C—H⋯O hydrogen bond is observed. In the crystal, pairs of C—H⋯π inter­actions link the mol­ecules, forming inversion dimers.

## Related literature

For electrophilic aromatic aroylation of the 2,7-dimeth­oxy­naphthalene core, see: Okamoto & Yonezawa (2009 ▶); Okamoto *et al.* (2011 ▶).
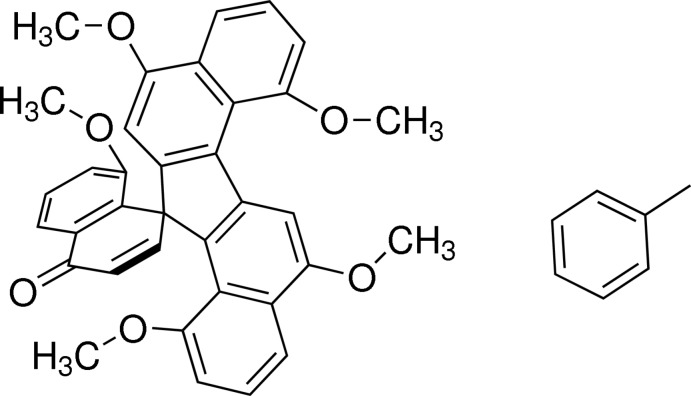


## Experimental

### 

#### Crystal data

C_35_H_28_O_6_·C_7_H_8_
*M* *_r_* = 636.71
Monoclinic, 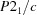

*a* = 12.4106 (6) Å
*b* = 12.4974 (7) Å
*c* = 21.4941 (11) Å
β = 97.319 (3)°
*V* = 3306.6 (3) Å^3^
*Z* = 4
Cu *K*α radiation
μ = 0.68 mm^−1^
*T* = 193 K
0.40 × 0.30 × 0.20 mm

#### Data collection

Rigaku R-AXIS RAPID diffractometer
Absorption correction: numerical (*NUMABS*; Higashi, 1999 ▶) *T*_min_ = 0.773, *T*_max_ = 0.876
48937 measured reflections
6036 independent reflections
3696 reflections with *I* > 2σ(*I*)
*R*_int_ = 0.087

#### Refinement

*R*[*F* ^2^ > 2σ(*F* ^2^)] = 0.043
*wR*(*F* ^2^) = 0.124
*S* = 0.96
6036 reflections
440 parameters
H-atom parameters constrained
Δρ_max_ = 0.20 e Å^−3^
Δρ_min_ = −0.18 e Å^−3^

### 

Data collection: *PROCESS-AUTO* (Rigaku, 1998 ▶); cell refinement: *PROCESS-AUTO*; data reduction: *CrystalClear* (Rigaku/MSC, 2004 ▶); program(s) used to solve structure: *SIR2004* (Burla *et al.*, 2005 ▶); program(s) used to refine structure: *SHELXL97* (Sheldrick, 2008 ▶); molecular graphics: *ORTEPIII* (Burnett & Johnson, 1996 ▶); software used to prepare material for publication: *SHELXL97*.

## Supplementary Material

Crystal structure: contains datablock(s) I, global. DOI: 10.1107/S160053681203824X/rz2797sup1.cif

Structure factors: contains datablock(s) I. DOI: 10.1107/S160053681203824X/rz2797Isup2.hkl

Additional supplementary materials:  crystallographic information; 3D view; checkCIF report

## Figures and Tables

**Table 1:** Hydrogen-bond geometry (Å, °) *Cg*1 is the centroid of the C5–C10 ring.

*D*—H⋯*A*	*D*—H	H⋯*A*	*D*⋯*A*	*D*—H⋯*A*
C3—H3⋯O4	0.95	2.22	2.810 (2)	120
C28—H28⋯*Cg*1^i^	0.95	2.65	3.550 (2)	159

Symmetry code: (i) 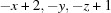.

**Fig. 1.**
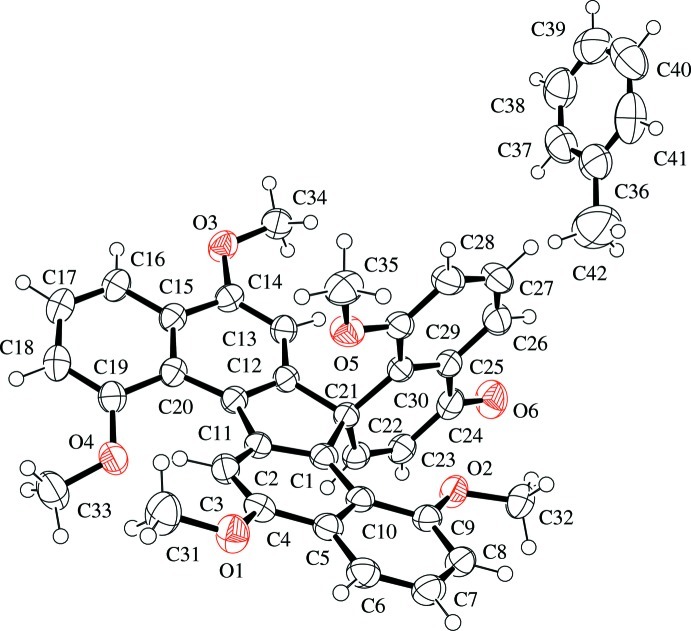
The molecular structure of the title compound, showing 50% probability displacement ellipsoids.

## supplementary crystallographic information

### Comment

In the course of our study on electrophilic aromatic aroylation of 2,7-dimethoxynaphthalene (Okamoto & Yonezawa, 2009; Okamoto *et al.*, 2011) and related compounds, we have found a unique trimerization reaction affording the title compound, C_35_H_28_O_6_.C_7_H_8_ (Fig. 1). The molecule is composed of dibenzo[*a*,*g*]fluorene and naphthalenone units originated from three naphthalene rings. The two units are connected by spiro bonding and configured in an approximately perpendicular fashion. The dihedral angle between the five-membered ring (C1/C2/C11/C12/C21) of the dibenzo[*a*,*g*]fluorene unit and the naphthalenone moiety (C21–C25/C30) is 87.51 (5)°. The dibenzo[*a*,*g*]fluorene unit is remarkably twisted, the dihedral angle between mean planes through the naphtlhalene ring systems [C1–C10 (*Nap1*) and C11–C20 (*Nap2*)] being 22.44 (3)°. This configuration presumably originates from the steric hindrance between the aromatic H3 atom of *Nap1* and the O4 methoxy group of *Nap2*. Between these atoms an intramolecular hydrogen bond is observed (Table 1). In the crystal packing, centrosymmetrically-related molecules are linked into dimers *via* C—H···π hydrogen interactions (Table 1).

### Experimental

1,5-Dimethoxynaphthalene (0.6 mmol), 1,3-dinitrobenzene (0.09 mmol) and CH_2_Cl_2_ (3 ml) were placed into a dried flask, followed by stirring at room temperature for 5 min under nitrogen atmosphere. To the reaction mixture, TiCl_4_ (3.0 mmol) was slowly added. The reaction mixture was poured into ice-cold water after it had been stirred at room temperature for 6 h. The aqueous layer was extracted with CHCl_3_ (40 ml). The combined extracts were washed with 2*M* aqueous NaOH followed by washing with brine. The organic layer thus obtained was dried over anhydrous MgSO_4_. The solvent was removed under reduced pressure to give the crude product (yield 99%), which was purified by preparative thin layer chromatography [toluene:EtOAc 40:1 *v*/*v*]. Transparent yellow single crystals suitable for X-ray diffraction were obtained by crystallization from toluene and hexane [1:1 *v*/*v*].

### Refinement

All H atoms were found in a difference Fourier map and refined as riding atoms, with C—H = 0.95 (aromatic), and 0.98 (methyl) Å, and with *U*_iso_(H) = 1.2*U*_eq_(C).

### Crystal data

**Table:**

C_35_H_28_O_6_·C_7_H_8_	*F*(000) = 1344
*M**_r_* = 636.71	*D*_x_ = 1.279 Mg m^−^^3^
Monoclinic, *P*2_1_/*c*	Cu *K*α radiation, λ = 1.54187 Å
Hall symbol: -P 2ybc	Cell parameters from 17422 reflections
*a* = 12.4106 (6) Å	θ = 3.5–68.3°
*b* = 12.4974 (7) Å	µ = 0.68 mm^−^^1^
*c* = 21.4941 (11) Å	*T* = 193 K
β = 97.319 (3)°	Block, yellow
*V* = 3306.6 (3) Å^3^	0.40 × 0.30 × 0.20 mm
*Z* = 4	

### Data collection

**Table:**

Rigaku R-AXIS RAPID diffractometer	6036 independent reflections
Radiation source: rotating anode	3696 reflections with *I* > 2σ(*I*)
Graphite monochromator	*R*_int_ = 0.087
Detector resolution: 10.000 pixels mm^-1^	θ_max_ = 68.2°, θ_min_ = 3.6°
ω scans	*h* = −14→14
Absorption correction: numerical (*NUMABS*; Higashi, 1999)	*k* = −14→15
*T*_min_ = 0.773, *T*_max_ = 0.876	*l* = −25→25
48937 measured reflections	

### Refinement

**Table:**

Refinement on *F*^2^	Secondary atom site location: difference Fourier map
Least-squares matrix: full	Hydrogen site location: inferred from neighbouring sites
*R*[*F*^2^ > 2σ(*F*^2^)] = 0.043	H-atom parameters constrained
*wR*(*F*^2^) = 0.124	*w* = 1/[σ^2^(*F*_o_^2^) + (0.0516*P*)^2^] where *P* = (*F*_o_^2^ + 2*F*_c_^2^)/3
*S* = 0.96	(Δ/σ)_max_ = 0.001
6036 reflections	Δρ_max_ = 0.20 e Å^−^^3^
440 parameters	Δρ_min_ = −0.18 e Å^−^^3^
0 restraints	Extinction correction: *SHELXL97* (Sheldrick, 2008), Fc^*^=kFc[1+0.001xFc^2^λ^3^/sin(2θ)]^-1/4^
Primary atom site location: structure-invariant direct methods	Extinction coefficient: 0.00363 (18)

### Special details

**Table:**

Geometry. All e.s.d.'s (except the e.s.d. in the dihedral angle between two l.s. planes) are estimated using the full covariance matrix. The cell e.s.d.'s are taken into account individually in the estimation of e.s.d.'s in distances, angles and torsion angles; correlations between e.s.d.'s in cell parameters are only used when they are defined by crystal symmetry. An approximate (isotropic) treatment of cell e.s.d.'s is used for estimating e.s.d.'s involving l.s. planes.
Refinement. Refinement of *F*^2^ against ALL reflections. The weighted *R*-factor *wR* and goodness of fit *S* are based on *F*^2^, conventional *R*-factors *R* are based on *F*, with *F* set to zero for negative *F*^2^. The threshold expression of *F*^2^ > σ(*F*^2^) is used only for calculating *R*-factors(gt) *etc*. and is not relevant to the choice of reflections for refinement. *R*-factors based on *F*^2^ are statistically about twice as large as those based on *F*, and *R*- factors based on ALL data will be even larger.

### Fractional atomic coordinates and isotropic or equivalent isotropic displacement parameters (Å^2^)

**Table:**

	*x*	*y*	*z*	*U*_iso_*/*U*_eq_	
O1	0.98979 (11)	−0.35612 (11)	0.34212 (7)	0.0490 (4)	
O2	0.95597 (10)	0.11986 (11)	0.32389 (7)	0.0437 (4)	
O3	0.39953 (10)	−0.00226 (11)	0.43477 (7)	0.0473 (4)	
O4	0.61527 (11)	−0.33814 (11)	0.29832 (7)	0.0485 (4)	
O5	0.83191 (10)	−0.04965 (12)	0.46869 (6)	0.0459 (4)	
O6	0.74754 (13)	0.36569 (12)	0.33675 (8)	0.0623 (5)	
C1	0.82886 (14)	−0.06441 (16)	0.33979 (8)	0.0335 (5)	
C2	0.77005 (14)	−0.15852 (16)	0.34250 (8)	0.0333 (5)	
C3	0.82219 (15)	−0.25873 (16)	0.34391 (8)	0.0375 (5)	
H3	0.7822	−0.3225	0.3483	0.045*	
C4	0.93038 (15)	−0.26346 (16)	0.33897 (9)	0.0370 (5)	
C5	0.99190 (15)	−0.16964 (16)	0.32933 (9)	0.0362 (5)	
C6	1.10248 (15)	−0.17731 (18)	0.31978 (9)	0.0420 (5)	
H6	1.1359	−0.2456	0.3192	0.050*	
C7	1.16078 (16)	−0.08835 (18)	0.31157 (9)	0.0454 (5)	
H7	1.2347	−0.0948	0.3048	0.054*	
C8	1.11359 (16)	0.01339 (18)	0.31294 (9)	0.0425 (5)	
H8	1.1557	0.0753	0.3074	0.051*	
C9	1.00695 (15)	0.02383 (16)	0.32228 (8)	0.0360 (5)	
C10	0.94035 (14)	−0.06757 (16)	0.33035 (8)	0.0338 (5)	
C11	0.65766 (14)	−0.13360 (16)	0.35384 (9)	0.0335 (5)	
C12	0.65255 (14)	−0.02529 (16)	0.36521 (8)	0.0334 (5)	
C13	0.56526 (14)	0.02494 (16)	0.38859 (9)	0.0366 (5)	
H13	0.5633	0.1005	0.3934	0.044*	
C14	0.48304 (14)	−0.03814 (17)	0.40435 (9)	0.0374 (5)	
C15	0.47837 (15)	−0.14984 (16)	0.39028 (9)	0.0365 (5)	
C16	0.39034 (15)	−0.21306 (17)	0.40481 (10)	0.0441 (5)	
H16	0.3354	−0.1819	0.4259	0.053*	
C17	0.38413 (16)	−0.31827 (18)	0.38869 (10)	0.0473 (6)	
H17	0.3272	−0.3610	0.4010	0.057*	
C18	0.46042 (16)	−0.36447 (17)	0.35417 (10)	0.0446 (5)	
H18	0.4534	−0.4374	0.3418	0.054*	
C19	0.54525 (15)	−0.30448 (17)	0.33820 (9)	0.0389 (5)	
C20	0.56338 (14)	−0.19760 (16)	0.36076 (9)	0.0362 (5)	
C21	0.75651 (14)	0.03203 (15)	0.35175 (8)	0.0319 (5)	
C22	0.72237 (14)	0.09293 (17)	0.29236 (9)	0.0380 (5)	
H22	0.7034	0.0525	0.2551	0.046*	
C23	0.71654 (15)	0.19880 (17)	0.28762 (10)	0.0410 (5)	
H23	0.6894	0.2305	0.2486	0.049*	
C24	0.75127 (16)	0.26731 (18)	0.34152 (10)	0.0442 (5)	
C25	0.79203 (15)	0.21481 (16)	0.40175 (10)	0.0385 (5)	
C26	0.82586 (17)	0.28005 (19)	0.45398 (11)	0.0510 (6)	
H26	0.8233	0.3557	0.4500	0.061*	
C27	0.86239 (17)	0.2340 (2)	0.51039 (11)	0.0546 (6)	
H27	0.8860	0.2781	0.5455	0.066*	
C28	0.86533 (16)	0.12371 (19)	0.51689 (10)	0.0486 (6)	
H28	0.8901	0.0925	0.5564	0.058*	
C29	0.83248 (14)	0.05930 (17)	0.46608 (9)	0.0376 (5)	
C30	0.79658 (14)	0.10423 (16)	0.40684 (9)	0.0341 (5)	
C31	0.93609 (18)	−0.45288 (17)	0.35525 (12)	0.0601 (7)	
H31A	0.9089	−0.4468	0.3959	0.072*	
H31B	0.9874	−0.5127	0.3564	0.072*	
H31C	0.8751	−0.4657	0.3224	0.072*	
C32	1.01937 (16)	0.21448 (16)	0.32372 (10)	0.0471 (6)	
H32A	1.0530	0.2178	0.2849	0.057*	
H32B	1.0762	0.2138	0.3598	0.057*	
H32C	0.9727	0.2771	0.3263	0.057*	
C33	0.59652 (18)	−0.43963 (18)	0.26869 (12)	0.0603 (7)	
H33A	0.6492	−0.4508	0.2390	0.072*	
H33B	0.5227	−0.4417	0.2462	0.072*	
H33C	0.6048	−0.4962	0.3005	0.072*	
C34	0.40275 (16)	0.10806 (16)	0.45303 (10)	0.0477 (6)	
H34A	0.4760	0.1262	0.4728	0.057*	
H34B	0.3506	0.1204	0.4829	0.057*	
H34C	0.3838	0.1530	0.4159	0.057*	
C35	0.87216 (17)	−0.09981 (19)	0.52663 (10)	0.0534 (6)	
H35A	0.8295	−0.0761	0.5594	0.064*	
H35B	0.9485	−0.0801	0.5383	0.064*	
H35C	0.8662	−0.1777	0.5220	0.064*	
C36	0.6690 (2)	0.60751 (19)	0.55973 (15)	0.0693 (7)	
C37	0.5588 (2)	0.62074 (19)	0.55166 (14)	0.0686 (7)	
H37	0.5199	0.6090	0.5112	0.082*	
C38	0.5035 (2)	0.6496 (2)	0.59848 (18)	0.0782 (8)	
H38	0.4268	0.6571	0.5906	0.094*	
C39	0.5543 (3)	0.6681 (2)	0.65627 (18)	0.0884 (10)	
H39	0.5139	0.6901	0.6887	0.106*	
C40	0.6641 (4)	0.6553 (2)	0.66847 (15)	0.0885 (10)	
H40	0.7003	0.6676	0.7095	0.106*	
C41	0.7230 (2)	0.6240 (2)	0.62027 (19)	0.0827 (9)	
H41	0.7993	0.6139	0.6285	0.099*	
C42	0.7292 (3)	0.5760 (3)	0.50638 (19)	0.1418 (16)	
H42A	0.6934	0.5142	0.4846	0.170*	
H42B	0.8042	0.5572	0.5227	0.170*	
H42C	0.7293	0.6360	0.4770	0.170*	

### Atomic displacement parameters (Å^2^)

**Table:**

	*U*^11^	*U*^22^	*U*^33^	*U*^12^	*U*^13^	*U*^23^
O1	0.0438 (8)	0.0383 (9)	0.0650 (10)	0.0040 (7)	0.0074 (7)	−0.0013 (8)
O2	0.0378 (8)	0.0389 (9)	0.0560 (9)	−0.0039 (6)	0.0116 (7)	0.0003 (7)
O3	0.0391 (8)	0.0442 (10)	0.0614 (10)	0.0000 (6)	0.0175 (7)	−0.0008 (7)
O4	0.0465 (8)	0.0435 (9)	0.0559 (10)	−0.0059 (7)	0.0087 (7)	−0.0140 (7)
O5	0.0532 (9)	0.0491 (10)	0.0341 (8)	0.0018 (7)	0.0004 (6)	0.0046 (7)
O6	0.0713 (11)	0.0374 (10)	0.0785 (12)	−0.0002 (8)	0.0111 (9)	0.0042 (9)
C1	0.0350 (10)	0.0386 (12)	0.0264 (10)	−0.0006 (9)	0.0022 (8)	0.0001 (9)
C2	0.0356 (11)	0.0357 (12)	0.0282 (11)	−0.0024 (9)	0.0028 (8)	−0.0010 (9)
C3	0.0402 (11)	0.0357 (13)	0.0360 (11)	−0.0040 (9)	0.0028 (9)	−0.0007 (9)
C4	0.0380 (11)	0.0386 (13)	0.0342 (11)	0.0048 (9)	0.0034 (9)	−0.0052 (10)
C5	0.0350 (11)	0.0424 (13)	0.0312 (11)	0.0000 (9)	0.0043 (8)	−0.0046 (10)
C6	0.0373 (11)	0.0469 (14)	0.0422 (12)	0.0041 (10)	0.0075 (9)	−0.0054 (11)
C7	0.0353 (11)	0.0573 (15)	0.0446 (13)	−0.0013 (11)	0.0096 (10)	−0.0034 (11)
C8	0.0388 (11)	0.0493 (14)	0.0406 (12)	−0.0057 (10)	0.0089 (9)	−0.0010 (10)
C9	0.0370 (11)	0.0410 (13)	0.0306 (11)	−0.0013 (9)	0.0060 (9)	−0.0004 (10)
C10	0.0336 (10)	0.0414 (13)	0.0263 (10)	−0.0021 (9)	0.0037 (8)	−0.0014 (9)
C11	0.0319 (10)	0.0374 (12)	0.0306 (11)	−0.0023 (8)	0.0014 (8)	0.0012 (9)
C12	0.0301 (10)	0.0385 (12)	0.0306 (11)	−0.0030 (9)	0.0000 (8)	0.0002 (9)
C13	0.0363 (11)	0.0334 (12)	0.0398 (12)	0.0003 (9)	0.0038 (9)	0.0013 (9)
C14	0.0294 (10)	0.0430 (13)	0.0397 (12)	0.0023 (9)	0.0044 (9)	0.0033 (10)
C15	0.0327 (10)	0.0397 (13)	0.0363 (11)	−0.0016 (9)	0.0016 (9)	0.0031 (10)
C16	0.0358 (11)	0.0450 (14)	0.0514 (14)	−0.0030 (10)	0.0052 (10)	0.0053 (11)
C17	0.0399 (12)	0.0448 (14)	0.0567 (15)	−0.0080 (10)	0.0044 (10)	0.0090 (12)
C18	0.0421 (12)	0.0382 (13)	0.0510 (13)	−0.0059 (10)	−0.0041 (10)	0.0039 (11)
C19	0.0344 (11)	0.0388 (13)	0.0420 (12)	−0.0014 (9)	−0.0016 (9)	0.0002 (10)
C20	0.0337 (11)	0.0376 (12)	0.0363 (11)	−0.0019 (9)	0.0012 (9)	0.0034 (9)
C21	0.0311 (10)	0.0330 (12)	0.0316 (11)	−0.0017 (8)	0.0038 (8)	0.0009 (9)
C22	0.0329 (11)	0.0456 (14)	0.0355 (12)	−0.0034 (9)	0.0039 (9)	0.0012 (10)
C23	0.0395 (11)	0.0428 (14)	0.0404 (12)	−0.0014 (10)	0.0038 (9)	0.0086 (10)
C24	0.0380 (12)	0.0382 (14)	0.0581 (15)	−0.0024 (10)	0.0131 (10)	0.0022 (12)
C25	0.0366 (11)	0.0367 (13)	0.0436 (13)	−0.0042 (9)	0.0105 (9)	−0.0033 (10)
C26	0.0495 (13)	0.0457 (15)	0.0593 (16)	−0.0071 (11)	0.0130 (11)	−0.0127 (12)
C27	0.0550 (14)	0.0658 (18)	0.0433 (14)	−0.0107 (12)	0.0068 (11)	−0.0192 (13)
C28	0.0450 (12)	0.0621 (17)	0.0388 (13)	−0.0032 (11)	0.0057 (10)	−0.0069 (12)
C29	0.0320 (10)	0.0453 (14)	0.0358 (12)	−0.0020 (9)	0.0057 (9)	−0.0040 (10)
C30	0.0274 (10)	0.0395 (13)	0.0361 (11)	−0.0022 (9)	0.0070 (8)	−0.0045 (10)
C31	0.0607 (15)	0.0394 (15)	0.0811 (18)	0.0034 (11)	0.0128 (13)	0.0049 (13)
C32	0.0475 (12)	0.0417 (14)	0.0526 (14)	−0.0085 (10)	0.0080 (10)	0.0072 (11)
C33	0.0546 (14)	0.0498 (16)	0.0761 (17)	−0.0024 (11)	0.0068 (12)	−0.0210 (14)
C34	0.0445 (12)	0.0442 (15)	0.0563 (14)	0.0044 (10)	0.0130 (10)	−0.0042 (11)
C35	0.0517 (13)	0.0665 (17)	0.0408 (13)	0.0010 (11)	0.0005 (10)	0.0137 (12)
C36	0.082 (2)	0.0468 (17)	0.083 (2)	−0.0064 (14)	0.0237 (17)	−0.0103 (15)
C37	0.083 (2)	0.0510 (17)	0.0682 (19)	−0.0048 (14)	−0.0024 (16)	−0.0049 (14)
C38	0.0738 (19)	0.0569 (19)	0.105 (3)	−0.0048 (14)	0.0179 (19)	−0.0036 (18)
C39	0.131 (3)	0.054 (2)	0.088 (3)	−0.011 (2)	0.046 (2)	0.0019 (18)
C40	0.147 (3)	0.0517 (19)	0.060 (2)	−0.015 (2)	−0.016 (2)	0.0054 (15)
C41	0.0654 (18)	0.0530 (19)	0.124 (3)	−0.0079 (14)	−0.0097 (19)	0.0057 (19)
C42	0.177 (4)	0.098 (3)	0.171 (4)	−0.016 (3)	0.104 (3)	−0.037 (3)

### Geometric parameters (Å, º)

**Table:**

O1—C4	1.370 (2)	C21—C30	1.521 (2)
O1—C31	1.426 (2)	C22—C23	1.328 (3)
O2—C9	1.359 (2)	C22—H22	0.9500
O2—C32	1.421 (2)	C23—C24	1.461 (3)
O3—C14	1.370 (2)	C23—H23	0.9500
O3—C34	1.433 (2)	C24—C25	1.482 (3)
O4—C19	1.362 (2)	C25—C30	1.387 (3)
O4—C33	1.425 (2)	C25—C26	1.407 (3)
O5—C29	1.363 (2)	C26—C27	1.366 (3)
O5—C35	1.426 (2)	C26—H26	0.9500
O6—C24	1.234 (2)	C27—C28	1.386 (3)
C1—C2	1.389 (2)	C27—H27	0.9500
C1—C10	1.424 (2)	C28—C29	1.376 (3)
C1—C21	1.544 (3)	C28—H28	0.9500
C2—C3	1.408 (2)	C29—C30	1.411 (3)
C2—C11	1.479 (2)	C31—H31A	0.9800
C3—C4	1.362 (2)	C31—H31B	0.9800
C3—H3	0.9500	C31—H31C	0.9800
C4—C5	1.429 (3)	C32—H32A	0.9800
C5—C6	1.417 (2)	C32—H32B	0.9800
C5—C10	1.428 (3)	C32—H32C	0.9800
C6—C7	1.350 (3)	C33—H33A	0.9800
C6—H6	0.9500	C33—H33B	0.9800
C7—C8	1.402 (3)	C33—H33C	0.9800
C7—H7	0.9500	C34—H34A	0.9800
C8—C9	1.370 (2)	C34—H34B	0.9800
C8—H8	0.9500	C34—H34C	0.9800
C9—C10	1.433 (3)	C35—H35A	0.9800
C11—C12	1.378 (3)	C35—H35B	0.9800
C11—C20	1.441 (2)	C35—H35C	0.9800
C12—C13	1.400 (2)	C36—C37	1.366 (4)
C12—C21	1.535 (2)	C36—C41	1.401 (4)
C13—C14	1.366 (2)	C36—C42	1.499 (4)
C13—H13	0.9500	C37—C38	1.338 (4)
C14—C15	1.428 (3)	C37—H37	0.9500
C15—C16	1.415 (3)	C38—C39	1.340 (4)
C15—C20	1.429 (3)	C38—H38	0.9500
C16—C17	1.359 (3)	C39—C40	1.363 (4)
C16—H16	0.9500	C39—H39	0.9500
C17—C18	1.400 (3)	C40—C41	1.398 (4)
C17—H17	0.9500	C40—H40	0.9500
C18—C19	1.371 (3)	C41—H41	0.9500
C18—H18	0.9500	C42—H42A	0.9800
C19—C20	1.429 (3)	C42—H42B	0.9800
C21—C22	1.500 (3)	C42—H42C	0.9800
			
C4—O1—C31	117.67 (16)	C24—C23—H23	119.5
C9—O2—C32	118.37 (15)	O6—C24—C23	120.9 (2)
C14—O3—C34	116.71 (15)	O6—C24—C25	121.3 (2)
C19—O4—C33	118.36 (16)	C23—C24—C25	117.83 (19)
C29—O5—C35	118.16 (16)	C30—C25—C26	120.6 (2)
C2—C1—C10	120.50 (18)	C30—C25—C24	121.07 (19)
C2—C1—C21	109.51 (16)	C26—C25—C24	118.3 (2)
C10—C1—C21	129.90 (17)	C27—C26—C25	119.7 (2)
C1—C2—C3	120.75 (17)	C27—C26—H26	120.1
C1—C2—C11	109.89 (17)	C25—C26—H26	120.1
C3—C2—C11	128.60 (17)	C26—C27—C28	120.6 (2)
C4—C3—C2	119.47 (18)	C26—C27—H27	119.7
C4—C3—H3	120.3	C28—C27—H27	119.7
C2—C3—H3	120.3	C29—C28—C27	120.0 (2)
C3—C4—O1	124.25 (18)	C29—C28—H28	120.0
C3—C4—C5	121.75 (18)	C27—C28—H28	120.0
O1—C4—C5	114.00 (17)	O5—C29—C28	123.67 (19)
C6—C5—C10	120.44 (18)	O5—C29—C30	115.60 (17)
C6—C5—C4	120.73 (18)	C28—C29—C30	120.7 (2)
C10—C5—C4	118.82 (17)	C25—C30—C29	118.22 (18)
C7—C6—C5	120.6 (2)	C25—C30—C21	121.58 (17)
C7—C6—H6	119.7	C29—C30—C21	120.06 (18)
C5—C6—H6	119.7	O1—C31—H31A	109.5
C6—C7—C8	120.79 (19)	O1—C31—H31B	109.5
C6—C7—H7	119.6	H31A—C31—H31B	109.5
C8—C7—H7	119.6	O1—C31—H31C	109.5
C9—C8—C7	120.23 (19)	H31A—C31—H31C	109.5
C9—C8—H8	119.9	H31B—C31—H31C	109.5
C7—C8—H8	119.9	O2—C32—H32A	109.5
O2—C9—C8	123.33 (18)	O2—C32—H32B	109.5
O2—C9—C10	115.04 (16)	H32A—C32—H32B	109.5
C8—C9—C10	121.63 (19)	O2—C32—H32C	109.5
C1—C10—C5	118.16 (17)	H32A—C32—H32C	109.5
C1—C10—C9	125.50 (18)	H32B—C32—H32C	109.5
C5—C10—C9	116.33 (17)	O4—C33—H33A	109.5
C12—C11—C20	118.07 (17)	O4—C33—H33B	109.5
C12—C11—C2	107.55 (16)	H33A—C33—H33B	109.5
C20—C11—C2	134.06 (18)	O4—C33—H33C	109.5
C11—C12—C13	124.01 (17)	H33A—C33—H33C	109.5
C11—C12—C21	111.47 (16)	H33B—C33—H33C	109.5
C13—C12—C21	124.46 (18)	O3—C34—H34A	109.5
C14—C13—C12	117.84 (19)	O3—C34—H34B	109.5
C14—C13—H13	121.1	H34A—C34—H34B	109.5
C12—C13—H13	121.1	O3—C34—H34C	109.5
C13—C14—O3	124.34 (19)	H34A—C34—H34C	109.5
C13—C14—C15	121.55 (18)	H34B—C34—H34C	109.5
O3—C14—C15	114.11 (17)	O5—C35—H35A	109.5
C16—C15—C14	120.71 (18)	O5—C35—H35B	109.5
C16—C15—C20	119.95 (19)	H35A—C35—H35B	109.5
C14—C15—C20	119.34 (17)	O5—C35—H35C	109.5
C17—C16—C15	120.3 (2)	H35A—C35—H35C	109.5
C17—C16—H16	119.9	H35B—C35—H35C	109.5
C15—C16—H16	119.9	C37—C36—C41	116.9 (3)
C16—C17—C18	120.9 (2)	C37—C36—C42	121.6 (3)
C16—C17—H17	119.5	C41—C36—C42	121.5 (3)
C18—C17—H17	119.5	C38—C37—C36	122.6 (3)
C19—C18—C17	120.0 (2)	C38—C37—H37	118.7
C19—C18—H18	120.0	C36—C37—H37	118.7
C17—C18—H18	120.0	C37—C38—C39	121.2 (3)
O4—C19—C18	124.07 (19)	C37—C38—H38	119.4
O4—C19—C20	114.62 (17)	C39—C38—H38	119.4
C18—C19—C20	121.25 (19)	C38—C39—C40	119.9 (3)
C19—C20—C15	116.60 (17)	C38—C39—H39	120.0
C19—C20—C11	125.36 (18)	C40—C39—H39	120.0
C15—C20—C11	117.98 (18)	C39—C40—C41	119.6 (3)
C22—C21—C30	112.98 (16)	C39—C40—H40	120.2
C22—C21—C12	104.13 (14)	C41—C40—H40	120.2
C30—C21—C12	109.23 (14)	C40—C41—C36	119.7 (3)
C22—C21—C1	111.19 (15)	C40—C41—H41	120.1
C30—C21—C1	116.95 (15)	C36—C41—H41	120.1
C12—C21—C1	100.82 (15)	C36—C42—H42A	109.5
C23—C22—C21	125.26 (19)	C36—C42—H42B	109.5
C23—C22—H22	117.4	H42A—C42—H42B	109.5
C21—C22—H22	117.4	C36—C42—H42C	109.5
C22—C23—C24	121.0 (2)	H42A—C42—H42C	109.5
C22—C23—H23	119.5	H42B—C42—H42C	109.5
			
C10—C1—C2—C3	8.0 (3)	O4—C19—C20—C11	−11.6 (3)
C21—C1—C2—C3	−169.04 (16)	C18—C19—C20—C11	171.08 (18)
C10—C1—C2—C11	178.89 (16)	C16—C15—C20—C19	9.9 (3)
C21—C1—C2—C11	1.8 (2)	C14—C15—C20—C19	−169.58 (17)
C1—C2—C3—C4	−3.7 (3)	C16—C15—C20—C11	−172.52 (17)
C11—C2—C3—C4	−172.72 (17)	C14—C15—C20—C11	8.0 (3)
C2—C3—C4—O1	177.48 (17)	C12—C11—C20—C19	165.59 (18)
C2—C3—C4—C5	−3.1 (3)	C2—C11—C20—C19	−21.8 (3)
C31—O1—C4—C3	−4.0 (3)	C12—C11—C20—C15	−11.7 (3)
C31—O1—C4—C5	176.49 (17)	C2—C11—C20—C15	160.88 (19)
C3—C4—C5—C6	−175.38 (18)	C11—C12—C21—C22	107.55 (18)
O1—C4—C5—C6	4.1 (3)	C13—C12—C21—C22	−74.9 (2)
C3—C4—C5—C10	5.5 (3)	C11—C12—C21—C30	−131.50 (17)
O1—C4—C5—C10	−175.01 (16)	C13—C12—C21—C30	46.0 (2)
C10—C5—C6—C7	0.1 (3)	C11—C12—C21—C1	−7.76 (19)
C4—C5—C6—C7	−179.06 (18)	C13—C12—C21—C1	169.75 (17)
C5—C6—C7—C8	0.6 (3)	C2—C1—C21—C22	−106.63 (17)
C6—C7—C8—C9	−0.4 (3)	C10—C1—C21—C22	76.7 (2)
C32—O2—C9—C8	−8.1 (3)	C2—C1—C21—C30	121.54 (17)
C32—O2—C9—C10	172.65 (15)	C10—C1—C21—C30	−55.2 (2)
C7—C8—C9—O2	−179.73 (18)	C2—C1—C21—C12	3.29 (18)
C7—C8—C9—C10	−0.5 (3)	C10—C1—C21—C12	−173.41 (17)
C2—C1—C10—C5	−5.4 (3)	C30—C21—C22—C23	−6.2 (2)
C21—C1—C10—C5	170.98 (17)	C12—C21—C22—C23	112.2 (2)
C2—C1—C10—C9	175.28 (17)	C1—C21—C22—C23	−140.00 (19)
C21—C1—C10—C9	−8.3 (3)	C21—C22—C23—C24	4.2 (3)
C6—C5—C10—C1	179.67 (17)	C22—C23—C24—O6	178.75 (19)
C4—C5—C10—C1	−1.2 (3)	C22—C23—C24—C25	−0.6 (3)
C6—C5—C10—C9	−0.9 (3)	O6—C24—C25—C30	−179.69 (18)
C4—C5—C10—C9	178.19 (16)	C23—C24—C25—C30	−0.3 (3)
O2—C9—C10—C1	−0.2 (3)	O6—C24—C25—C26	0.5 (3)
C8—C9—C10—C1	−179.49 (18)	C23—C24—C25—C26	179.91 (17)
O2—C9—C10—C5	−179.56 (15)	C30—C25—C26—C27	−0.7 (3)
C8—C9—C10—C5	1.2 (3)	C24—C25—C26—C27	179.06 (18)
C1—C2—C11—C12	−6.9 (2)	C25—C26—C27—C28	−0.7 (3)
C3—C2—C11—C12	163.06 (19)	C26—C27—C28—C29	0.8 (3)
C1—C2—C11—C20	180.0 (2)	C35—O5—C29—C28	−2.9 (3)
C3—C2—C11—C20	−10.1 (3)	C35—O5—C29—C30	177.09 (15)
C20—C11—C12—C13	6.1 (3)	C27—C28—C29—O5	−179.35 (18)
C2—C11—C12—C13	−168.34 (17)	C27—C28—C29—C30	0.7 (3)
C20—C11—C12—C21	−176.39 (15)	C26—C25—C30—C29	2.1 (3)
C2—C11—C12—C21	9.2 (2)	C24—C25—C30—C29	−177.71 (16)
C11—C12—C13—C14	3.8 (3)	C26—C25—C30—C21	177.65 (16)
C21—C12—C13—C14	−173.39 (17)	C24—C25—C30—C21	−2.2 (3)
C12—C13—C14—O3	171.61 (17)	O5—C29—C30—C25	177.95 (16)
C12—C13—C14—C15	−7.9 (3)	C28—C29—C30—C25	−2.1 (3)
C34—O3—C14—C13	−2.6 (3)	O5—C29—C30—C21	2.3 (2)
C34—O3—C14—C15	176.89 (17)	C28—C29—C30—C21	−177.68 (16)
C13—C14—C15—C16	−177.56 (18)	C22—C21—C30—C25	5.0 (2)
O3—C14—C15—C16	2.9 (3)	C12—C21—C30—C25	−110.42 (19)
C13—C14—C15—C20	2.0 (3)	C1—C21—C30—C25	135.98 (18)
O3—C14—C15—C20	−177.56 (16)	C22—C21—C30—C29	−179.54 (15)
C14—C15—C16—C17	177.03 (19)	C12—C21—C30—C29	65.1 (2)
C20—C15—C16—C17	−2.5 (3)	C1—C21—C30—C29	−48.5 (2)
C15—C16—C17—C18	−4.0 (3)	C41—C36—C37—C38	1.0 (4)
C16—C17—C18—C19	2.5 (3)	C42—C36—C37—C38	−179.5 (3)
C33—O4—C19—C18	3.3 (3)	C36—C37—C38—C39	0.6 (4)
C33—O4—C19—C20	−173.86 (17)	C37—C38—C39—C40	−1.5 (4)
C17—C18—C19—O4	−171.42 (18)	C38—C39—C40—C41	0.7 (4)
C17—C18—C19—C20	5.6 (3)	C39—C40—C41—C36	0.9 (4)
O4—C19—C20—C15	165.71 (16)	C37—C36—C41—C40	−1.7 (4)
C18—C19—C20—C15	−11.6 (3)	C42—C36—C41—C40	178.8 (3)

### Hydrogen-bond geometry (Å, º)

Cg1 is the centroid of the C5–C10 ring.

**Table:**

*D*—H···*A*	*D*—H	H···*A*	*D*···*A*	*D*—H···*A*
C3—H3···O4	0.95	2.22	2.810 (2)	120
C28—H28···*Cg*1^i^	0.95	2.65	3.550 (2)	159

Symmetry code: (i) −*x*+2, −*y*, −*z*+1.
